# The impact of neurological and cerebellar soft signs on psychosocial functioning in bipolar disorder

**DOI:** 10.3389/fpsyt.2025.1632857

**Published:** 2025-07-30

**Authors:** Adrian Andrzej Chrobak, Zbigniew Soltys, Anna Starowicz-Filip, Krzysztof Styczeń, Małgorzata Dec-Ćwiek, Dominika Dudek, Marcin Siwek

**Affiliations:** ^1^ Department of Adult Psychiatry, Jagiellonian University Medical College, Cracow, Poland; ^2^ Laboratory of Experimental Neuropathology, Institute of Zoology and Biomedical Research, Jagiellonian University, Cracow, Poland; ^3^ Department of Medical Psychology, Jagiellonian University Medical College, Kraków, Poland; ^4^ Department of Neurosurgery, University Hospital in Krakow, Kraków, Poland; ^5^ Department of Neurology, Jagiellonian University, Medical College, Krakow, Poland; ^6^ Department of Neurology, University Hospital, Kraków, Poland; ^7^ Department of Affective Disorders, Jagiellonian University Medical College, Cracow, Poland

**Keywords:** cerebellum, affective disorders, neurology, movement disorders, bipolar disorder

## Abstract

**Background:**

Patients with bipolar disorder (BD) present motor dysfunctions in the form of neurological and cerebellar soft signs (NSS and CSS, respectively). Little is known about the clinical utility of these symptoms and their impact on patients’ psychosocial functioning. The aim of our study is to assess the relationships between severity of NSS and CSS, as well as various dimensions of the daily functioning of patients with BD.

**Methods:**

A total of 100 participants were enrolled to this study: 60 patients with euthymic BD and 40 healthy controls (HC). Psychosocial functioning was evaluated with the use of Functioning Assessment Short Test (FAST) total score and its subscales. NSS were assessed with the use of the Neurological Evaluation Scale (NES). CSS were measured with the International Co-operative Ataxia Rating Scale (ICARS).

**Results:**

General psychosocial functioning was decreased by CSS and NSS severity represented by total NES and ICARS scores, as well as by higher measures of kinetic functions, sensory integration, motor coordination, and speech disorders subscales. Patients’ autonomy rates were decreased by total ICARS, kinetic functions, and speech disorders scores. Occupational functioning was limited by the majority of CSS and NSS measures. Cognitive functioning was associated with motor coordination impairments. Leisure time activities were influenced by total CSS severity and kinetic dysfunctions. We have shown that the severity of both CSS and NSS is a full mediator of the associations between duration of treatment and general psychosocial functioning.

**Conclusions:**

Our results suggest that even “soft” neurological abnormalities may have an impact on the psychosocial functioning of patients with BD.

## 1 Introduction

Neurological impairments have been described in the group of mentally ill patients since the time of Kraepelin (1). Initially, the majority of studies focused on individuals diagnosed with schizophrenia. It has been demonstrated that these patients exhibit a heterogeneous group of symptoms including motor coordination impairments, oculomotor deficits, tremors, and frontal release responses (2–4). These motor abnormalities were described as fleeting, ambiguous, and difficult to interpret. Therefore, they have been termed neurological soft signs (NSS), indicating the challenge of associating them with specific central nervous system deficits (5). However, with the advancement of neuroimaging research, this name has become obsolete as the neural underpinnings of those symptoms have been recognized. In a thorough metanalysis, Zhao et al. (6) have shown that NSS are associated with the impairments of the prefrontal-thalamo-cerebellar brain network (6). Studies have indicated that the cerebellum, which is linked to these symptoms, also plays a role in the neurobiology of various mental disorders (7). In order to measure the set of neurological symptoms specifically associated with cerebellar deficits, Varambally et al. (8) introduced the term “cerebellar soft signs” (CSS) that incorporates specific impairments of posture, kinetic functions, limb ataxia, dysarthria, and oculomotor deficits (8). A growing number of studies have shown relationships between neurological abnormalities, cognitive impairments, temperamental traits, psychopathology, and structural brain abnormalities, indicating that these symptoms may constitute an easy-to-assess biological marker (2–4, 9, 10). CSS and NSS are not exclusive to schizophrenia, as these symptoms have also been observed in patients with BD. This may be due to the substantial overlap between the two disorders in genetics, neurophysiology, and neuropathology (10, 11). There is a hypothesis that NSS could serve as a common intermediate phenotype in both schizophrenia and bipolar disorder (BD), indicating a shared neurodevelopmental and genetic deficit (10). In comparison to schizophrenia research, there are significantly fewer publications addressing neurological impairments in BD. It has been demonstrated that patients with BD exhibit markedly more severe NSS and CSS than healthy controls (HC) (11, 12). Additionally, these patients present moderately less severe NSS compared to those with schizophrenia (13). Our studies have indicated comparable levels of CSS severity between the two disorders (11, 12, 14).

There is limited knowledge regarding the clinical correlates of neurological symptoms in patients with BD. Some studies suggest that the severity of NSS may be associated with psychosocial functioning. Goswami et al. (15) showed that there is a strong correlation between neurological abnormalities and marked social disability in patients with BD (15). Negash et al. (16) revealed that NSS total score was associated with poor quality of dwelling in the group of euthymic BD I patients (16). Baş et al. (17) demonstrated a significant correlation between NSS score and functionality, as well as the executive functions of patients with euthymic BD. The low-functioning group presented more severe neurological abnormalities; however, in a regression model, NSS total score did not predict the level of patients’ functioning (17). Valerio et al. (18) have shown that NSS are negatively correlated with premorbid IQ as well as with performance in attention, executive functions, and language (18). Authors demonstrated that although NSS severity was related to poor functioning, when added to a multiple regression model including neurocognition, neurological symptoms had no significant impact on psychosocial functioning. Mrad et al. (19) showed significant associations between NSS severity and global assessment of functioning scores as well as lower school level (19). Our recent study has shown that both NSS and CSS are more severe in the late stage of BD in comparison to the early phases of this disorder. In the case of CSS, there are no studies evaluating associations between those neurological abnormalities and psychosocial functioning in patients with BD.

The aim of this study is to evaluate the relationships between the severity of both NSS and CSS and various aspects of the daily functioning of patients with euthymic BD. We hypothesized that higher rates of both groups of neurological abnormalities will be associated with lower levels of psychosocial functioning.

## 2 Methods

### 2.1 Participants

In this study, we have recruited 100 participants: 60 patients with euthymic BD (BD I, BD II, and history of psychotic symptoms) and 40 HC. Patients were diagnosed according to ICD-10 and DSM-5 criteria, based on consensus of two experienced psychiatrists. Patients with BD were recruited to the study if they met the following inclusion criteria: (a) euthymia, determined according to the International Society for Bipolar Disorders (ISBD) Task Force (20) [8 points or less on the Bipolar Depression Rating Scale (21) and 8 points or less on the Young Mania Rating Scale (22)]; and (b) treatment with the use of the antipsychotic drugs from the group of dibenzoxazepines (clozapine, quetiapine, or olanzapine), in order to minimize the drug’s effects on patient’s motor performance and to achieve a relatively homogeneous group of patients in terms of side effect profile. Treatment with the use of valproic acid and lamotrigine was also accepted. Patients were excluded from the study if they met the following criteria (1): lithium treatment, since it may affect cerebellar functions (23) (2); alcohol or drug abuse diagnosis according to the substance use disorder of DSM-5 criteria (3); chronic, severe, or acute somatic or neurological disease; and (4) treatment other than mentioned. None of the participants were under benzodiazepine treatment at the period of examination and interview.

HC consisted of mentally healthy individuals recruited from the authors’ social network. An experienced psychiatrist screened those participants with the use of the Mini-International Neuropsychiatric Interview. HC reported negative history of mental and neurological disorders and did not meet aforementioned exclusion criteria for patients. Demographic data of the studied groups have been summarized in **Table 1**. All participants signed a written consent prior to the assessment. The study was approved by the Jagiellonian University Bioethics Committee. The investigation was carried out in accordance with the latest version of the Declaration of Helsinki.

**Table 1:** The description of study groups.

	BD group	HC group	df	*t* or χ2	*p*	Cohen’s *d*
Age (years, mean ± SD)	37.48 ± 12.02	37.53 ± 8.59	98	−0.01893	0.985	0.003
Sex (male/female)	27/33	22/18	1	0.60191	0.437	–
Years of education (years, mean ± SD)	16.19 ± 1.83	15.775 ± 1.91	98	1.0959	0.276	0.22
Duration of treatment (years, mean ± SD)	10.91 ± 8.35	–	–	–	–	–
Equivalent of the daily dose of olanzapine (mg/day ± SD)	9.80 ± 5.16	–	–	–	–	–
History of psychotic symptoms (+/−)	15/60	–	–	–	–	–
BD type (I/II)	33/27	–	–	–	–	–

BD, bipolar disorder; HC, healthy controls; SD, standard deviation.

### 2.2 Psychosocial functioning assessment

Patients’ psychosocial functioning was evaluated during an interview with the use of the Functioning Assessment Short Test (FAST) scale (24). This clinical tool was developed for the evaluation of difficulties presented by patients with BD. The instrument consists of 24 items divided into six subscales representing different dimensions of functioning: autonomy, occupational functioning, cognitive functioning, financial issues, interpersonal relationships, and leisure time. Each individual item is scored from 0 to 3. The global score is calculated by summing the scores of each individual item, with higher scores indicating more severe difficulties. We have used a Polish version of FAST, supplied by one of the authors of this scale, Flavio Kapczinski.

### 2.3 Neurological assessment

Neurological examination was performed by the main author (A.A.C.) who was blind to the patients’ FAST scores, BD type, and history of psychotic symptoms.

NSS were assessed with the use of the Neurological Evaluation Scale (NES) (5). Based on our literature analysis, this clinical tool was selected as it is the most widely utilized instrument for assessing NSS in psychiatric research and for evaluating neurological abnormalities in patients with BD (25). NES consists of 26 items divided into four subscales: motor coordination, sequencing of complex motor acts, sensory integration, and other symptoms. Symptoms were rated according to the standardized instructions.

CSS were assessed with the use of the International Co-operative Ataxia Rating Scale (ICARS) (26). The instrument consists of 19 items divided into four subscales: posture and gait disturbances, kinetic functions, speech disorders, and oculomotor disorders. Items were rated according to the standardized instructions. ICARS was validated in groups of patients with spinocerebellar atrophy 1, 2, 3, 6, 7, and 14, Friedreich ataxia, and multiple system atrophy (27).

### 2.4 Statistical analyses

Statistical analyses were performed with the use of R software (28). Student’s *t*-test and *χ* ^2^ tests were used to compare demographic characteristics. Shapiro–Wilk and Levene tests were used to evaluate the normality of distribution and homogeneity of variance. Associations between CSS, NSS (independent variables), and FAST (dependent variable) were calculated by a series of simple linear regression analyses. The same method was used to evaluate associations between neurological and clinical abnormalities along with demographic variables: age, years of education, duration of the treatment, age of onset (defined as age − duration of the treatment), and equivalent of the olanzapine daily dose. Daily dosages of antipsychotics were converted to equivalent of olanzapine according to Leucht et al. (2015) (29).

We have performed mediation analysis to evaluate the role of CSS and NSS severity (ICARS and NES total scores) in the relationship between duration of treatment and general psychosocial functioning (FAST total score). As the first step of the analysis, we have calculated the total effect that the independent variable (duration of treatment) has on the dependent variable (FAST total score) with the use of simple linear regression analysis. We have not disqualified pairs of variables with no statistically significant total effect from further mediation analysis, according to the interpretations indicating that no correlation does not disprove causation effect (30, 31). Subsequently, we have evaluated the effect of the independent variable (duration of treatment) on mediator (ICARS total score in the first mediation analysis and NES total score in the second one) with the use of simple linear regression. Next, we have evaluated the effect of the mediator on FAST total score (dependent variable) with the use of simple linear regression controlling for the effect of the independent variable (duration of treatment). Then, we have calculated average causal mediation effect (ACME—the effect of the mediator alone), average direct effect (ADE—the unmediated effect), and proportion mediated. ACME measures the indirect effect of duration of treatment on FAST score that goes through the mediator (NES and ICARS total scores). ADE shows the direct effect of the independent variable on the dependent one. Proportion mediated represents ACME divided by total effect. Each mediation analysis model was recalculated 1,000 times with random subsamples from the data.

Mediation analysis was carried out with the use of the “mediation” package (32). Other analysis was carried out with the use of functions from the stats package, and graphics were done with functions from the ggplot2 package.

## 3 Results

A comparison of NSS and CSS measures between BD and HC groups indicated that patients had significantly higher scores in every NES and ICARS category except for the speech disorders subscore. The BD group exhibited higher scores across all measures of the FAST, indicating inferior psychosocial functioning compared to the HC group. Detailed results of those comparisons are presented in **Table 2**.

**Table 2:** Comparison of NSS, CSS, and psychosocial functioning measures between the BD group and the HC group.

	BD group		HC group			df	*t*		*p*	Cohen’s *d*
	Mean	SD	Mean	SD						
Cerebellar soft signs										
ICARS total score	9.98	7.22	4.15	5.26		97.144		4.6643	<0.001	0.89
Posture and gait disturbances	3.58	2.47	1.25	1.56		97.792		5.778	<0.001	1.08
Kinetic functions	4.3	4.97	2.2	3.57		98		2.3046	0.02	0.47
Speech disorders	0.52	0.93	0.275	0.75		98		1.3721	0.17	0.28
Oculomotor disorders	1.57	1.84	0.425	0.81		87.193		4.219	<0.001	0.75
Neurological soft signs										
NES total score	19.57	10.84	9.7	8.45		98		4.8547	<0.001	0.99
Sensory integration	2.53	1.83	1.6	1.78		98		2.5282	0.01	0.516
Motor coordination	3.83	3.20	1.775	2.19		97.914		3.8185	<0.001	0.72
Sequencing of complex motor acts	4.45	3.42	2.375	2.79		98		3.19	0.002	0.65
Psychosocial functioning										
FAST total score	20.95	15.06	6.125	7.432388	91.509			6.52	<0.001	1.177
Autonomy	2.78	2.91	0.8	1.090754	81.023			4.795	<0.001	0.839
Occupational functioning	5	4.66	1.225	1.731866	80.754			5.713	<0.001	0.999
Cognitive functioning	5.2	3.87	1.425	2.110869	94.858			6.23	<0.001	1.139
Financial issues	1.82	2.05	0.3	0.7578647	80.618			5.229	<0.001	0.914
Interpersonal relationships	4.38	4.48	1.6	2.362094	93.792			4.0427	<0.001	0.735
Leisure time	1.8	1.71	0.775	1.208676	97.59			3.515	<0.001	0.67

BD, bipolar disorder; HC, healthy controls; FAST, Functioning Assessment Short Test; ICARS, International Co-operative Ataxia Rating Scale; NES, Neurological Evaluation Scale; SD, standard deviation. *t*-test with Welch correction.

**Figure 1:**
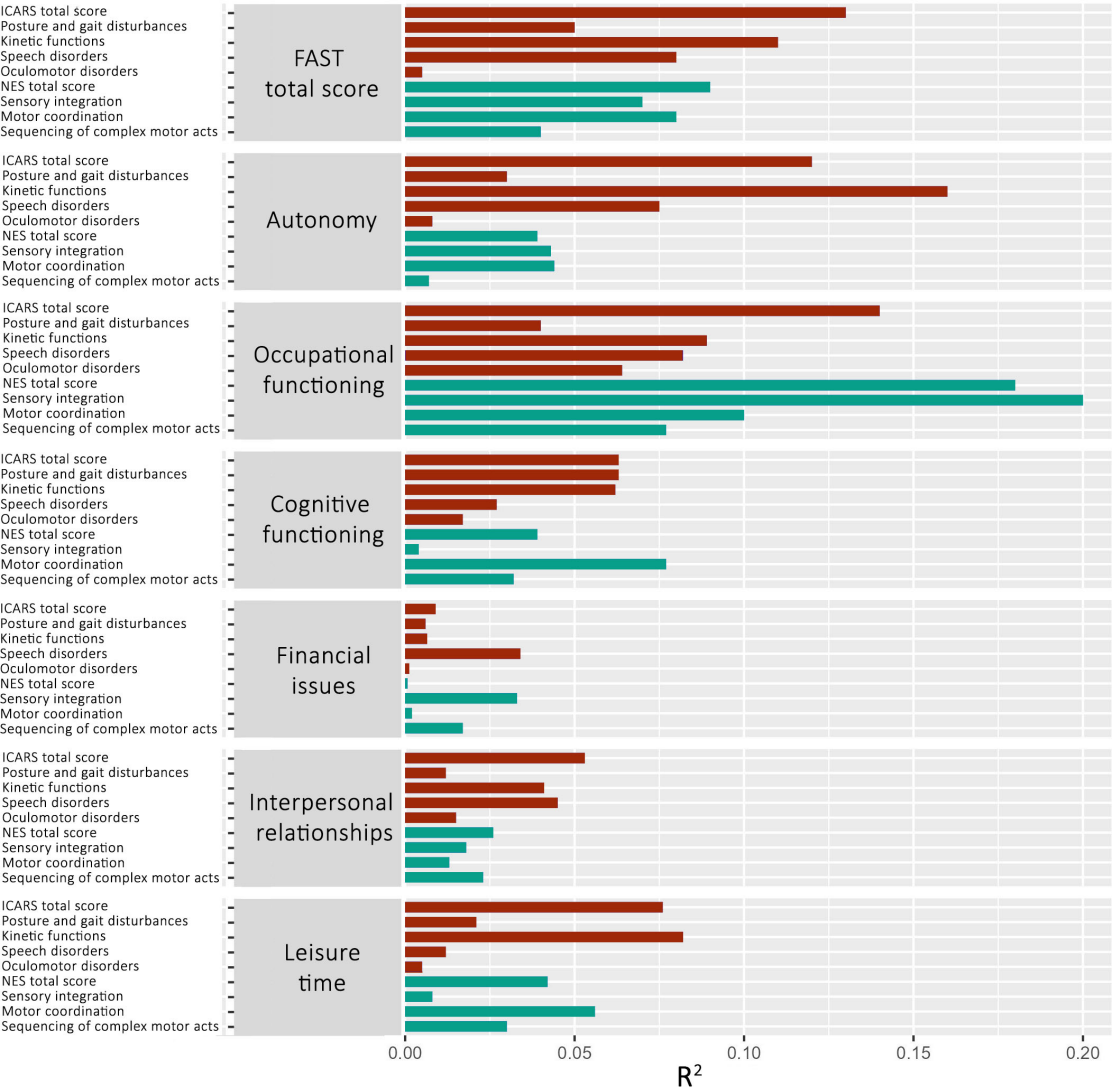
Associations between ICARS and NES scores (independent variable) and FAST scores (dependent variable) in the group of patients with BD. Bars represent *R* ^2^ measures of simple linear regressions between variables. FAST, Functioning Assessment Short Test; ICARS, International Co-operative Ataxia Rating Scale; NES, Neurological Evaluation Scale; red color, ICARS scores; turquoise color, NES scores.

Regression analyses revealed statistically significant associations between measures of neurological abnormalities and psychosocial functioning in patients with BD. General functional impairment reflected by total FAST score was increased by higher total ICARS, kinetic functions, speech disorders, NES total, sensory integration, and motor coordination scores. Autonomy rates were decreased by higher total ICARS, kinetic functions, and speech disorders scores. Occupational functioning impairments were associated with higher total ICARS, kinetic functions, speech disorders, total NES, sensory integration, sequencing of complex motor acts, and motor coordination scores. Poorer cognitive functioning was related to higher rates of motor coordination impairments. Leisure time activities scores were decreased by higher ICARS total and kinetic functions scores. Financial issues and interpersonal relationships were not influenced by ICARS and NES scores. Besides the relationships between kinetic functions, speech disorder scores, and occupational functioning, all of the previously mentioned associations were significantly stronger in the BD group when compared to HC. The control group showed no significant relationships between the severity of neurological abnormalities and FAST scores. Detailed results of these analyses are presented in **Figure 1**; **Table 3**.

**Table 3:** Regression analyses evaluating associations between NSS and CSS scores (independent variables) and psychosocial functioning measures (dependent variables) in the BD group and the HC group.

Independent variables	BD group				HC group				Difference between the β (*p*)
	Intercept	β	*R* ^2^	*p*	Intercept	β	*R* ^2^	*p*	
Total FAST score									
ICARS total score	13	0.75	0.13	0.005	7.7	−0.38	0.07	0.09	0.009
Posture and gait disturbances	16	1.3	0.05	0.09	7.3	−0.9	0.04	0.24	0.12
Kinetic functions	17	1	0.11	0.008	7	−0.4	0.04	0.23	0.03
Speech disorders	19	4.6	0.08	0.03	6.9	−2.7	0.08	0.08	0.02
Oculomotor disorders	20	0.55	0.005	0.61	7.2	−2.5	0.08	0.08	0.25
NES total score	13	0.41	0.09	0.02	7.7	−0.16	0.03	0.26	0.04
Sensory integration	15	2.2	0.07	0.04	7.9	−1.1	0.07	0.09	0.02
Motor coordination	16	1.3	0.08	0.03	6.9	−0.46	0.02	0.41	0.09
Sequencing of complex motor acts	17	0.86	0.04	0.13	6.9	−0.33	0.016	0.44	0.17
Autonomy									
ICARS total score	1.4	0.14	0.12	0.007	1.1	−0.06	0.086	0.07	0.01
Posture and gait disturbances	2	0.21	0.03	0.17	1.1	−0.21	0.09	0.06	0.12
Kinetic functions	1.8	0.23	0.16	0.001	0.93	−0.06	0.04	0.23	0.01
Speech disorders	2.3	0.86	0.075	0.03	0.89	−0.31	0.045	0.19	0.5
Oculomotor disorders	3	−0.14	0.008	0.49	0.96	−0.37	0.077	0.08	0.64
NES total score	1.7	0.053	0.039	0.13	0.97	−0.017	0.018	0.41	0.18
Sensory integration	1.9	0.33	0.043	0.11	0.8	-0.0016	0	0.99	0.22
Motor coordination	2	0.19	0.044	0.11	0.92	−0.068	0.019	0.4	0.19
Sequencing of complex motor acts	2.5	0.072	0.007	0.52	0.88	−0.033	0.007	0.61	0.52
Occupational functioning									
ICARS total score	2.6	0.24	0.14	0.003	1.5	−0.064	0.038	0.23	0.02
Posture and gait disturbances	2.6	0.38	0.04	0.13	1.4	−0.16	0.021	0.37	0.22
Kinetic functions	3.8	0.28	0.089	0.02	1.4	−0.078	0.026	0.32	0.06
Speech disorders	4.3	1.4	0.082	0.03	1.3	−0.39	0.028	0.3	0.06
Oculomotor disorders	4	0.64	0.064	0.05	1.3	−0.26	0.015	0.44	0.25
NES total score	1.5	0.18	0.18	<0.001	1.4	−0.017	0.007	0.61	0.01
Sensory integration	2.1	1.1	0.2	<0.001	1.6	−0.25	0.064	0.12	<0.001
Motor coordination	3.2	0.46	0.1	0.01	1.2	0.011	0.0002	0.93	0.14
Sequencing of complex motor acts	3.3	0.38	0.077	0.03	1.3	−0.051	0.007	0.62	0.09
Cognitive functioning									
ICARS total score	3.8	0.13	0.063	0.05	1.9	−0.1	0.067	0.11	0.04
Posture and gait disturbances	3.8	0.39	0.063	0.05	1.9	−0.27	0.042	0.21	0.08
Kinetic functions	4.3	0.19	0.062	0.05	1.6	−0.1	0.029	0.29	0.08
Speech disorders	4.8	0.68	0.027	0.21	1.6	−0.71	0.064	0.11	0.1
Oculomotor disorders	5.6	−0.28	0.017	0.31	1.8	−0.78	0.091	0.06	0.46
NES total score	3.8	0.07	0.039	0.13	2	−0.059	0.056	0.14	0.08
Sensory integration	4.8	0.14	0.004	0.63	2	−0.37	0.1	0.05	0.18
Motor coordination	3.9	0.34	0.077	0.03	1.6	−0.12	0.017	0.43	0.09
Sequencing of complex motor acts	4.3	0.2	0.032	0.17	1.7	−0.11	0.022	0.36	0.16
Financial issues									
ICARS total score	1.5	0.027	0.009	0.47	0.38	−0.018	0.016	0.43	0.44
Posture and gait disturbances	1.6	0.062	0.006	0.57	0.33	−0.021	0.002	0.79	0.66
Kinetic functions	1.7	0.033	0.0065	0.54	0.35	−0.023	0.012	0.51	0.52
Speech disorders	1.6	0.41	0.034	0.16	0.34	−0.15	0.022	0.36	0.19
Oculomotor disorders	1.9	−0.039	0.0012	0.79	0.35	−0.12	0.017	0.43	0.82
NES total score	1.7	0.005	0.0007	0.84	0.46	−0.017	0.035	0.25	0.57
Sensory integration	1.3	0.2	0.033	0.17	0.38	−0.05	0.014	0.47	0.18
Motor coordination	1.7	0.028	0.002	0.74	0.37	−0.039	0.013	0.49	0.63
Sequencing of complex motor acts	2.2	−0.077	0.017	0.33	0.44	−0.061	0.05	0.16	0.89
Interpersonal relationships									
ICARS total score	3	0.14	0.053	0.08	1.9	−0.082	0.033	0.26	0.09
Posture and gait disturbances	3.7	0.2	0.012	0.4	1.8	−0.13	0.007	0.61	0.46
Kinetic functions	3.6	0.18	0.041	0.12	1.8	−0.086	0.017	0.42	0.17
Speech disorders	3.9	1	0.045	0.1	1.8	−0.8	0.065	0.11	0.06
Oculomotor disorders	3.9	0.3	0.015	0.35	1.9	−0.63	0.047	0.18	0.24
NES total score	3.1	0.066	0.026	0.22	1.8	−0.022	0.006	0.63	0.3
Sensory integration	3.6	0.33	0.018	0.31	2.1	−0.31	0.055	0.15	0.14
Motor coordination	3.8	0.16	0.013	0.38	1.8	−0.12	0.011	0.51	0.38
Sequencing of complex motor acts	3.5	0.2	0.023	0.25	1.7	−0.023	0.0007	0.87	0.39
Leisure time									
ICARS total score	1.1	0.065	0.076	0.03	0.98	−0.05	0.048	0.17	0.03
Posture and gait disturbances	1.4	0.1	0.021	0.27	0.92	−0.11	0.021	0.37	0.23
Kinetic functions	1.4	0.099	0.082	0.03	0.89	−0.053	0.024	0.34	0.05
Speech disorders	1.7	0.2	0.012	0.41	0.88	−0.39	0.058	0.13	0.13
Oculomotor disorders	1.7	0.069	0.005	0.57	0.93	−0.36	0.057	0.14	0.19
NES total score	1.2	0.032	0.042	0.11	1	−0.028	0.037	0.23	0.08
Sensory integration	1.6	0.083	0.008	0.5	1	−0.15	0.049	0.17	0.19
Motor coordination	1.3	0.13	0.056	0.07	0.99	−0.12	0.05	0.17	0.05
Sequencing of complex motor acts	1.4	0.086	0.03	0.19	0.9	−0.052	0.014	0.47	0.19

BD, bipolar disorder; HC, healthy controls; FAST, Functioning Assessment Short Test; ICARS, International Co-operative Ataxia Rating Scale; NES, Neurological Evaluation Scale; SD, standard deviation.

Regression analyses examining the relationship between clinical and demographic variables and ICARS, NES, and FAST scores identified significant associations. Duration of the treatment was related to higher total ICARS, kinetic functions, and speech disorders scores, as well as to all of the NES scores. The duration of the treatment was correlated exclusively with one subscale of the FAST assessment, specifically occupational functioning. This measure was also influenced by the daily dose of olanzapine and the patient’s age. Age of onset was not associated with ICARS, NES, and FAST scores. Years of education were related to lower total ICARS, kinetic functions, and speech disorders scores. Equivalent of the daily dose of olanzapine was associated with higher scores of the NES sensory integration subscore; however, this relationship was on the verge of statistical significance. Detailed results of these analyses are presented in **Table 4**.

**Table 4:** Regression analyses evaluating associations between demographic and clinical variables (independent variables) and NSS, CSS, and FAST scores (dependent variables) in the group of patients with BD.

Dependent variables	Age				Years of education				Duration of treatment				Age of onset				Equivalent of the daily dose of olanzapine			
	Intercept	β	*R* ^2^	*p*	Intercept	β	*R* ^2^	*p*	Intercept	β	*R* ^2^	*p*	Intercept	β	*R* ^2^	*p*	Intercept	β	*R* ^2^	*p*
Cerebellar soft signs																				
ICARS total score	6.881	0.083	0.019	0.29	32.606	−1.397	0.125	0.006	7.001	0.273	0.1	0.014	11.552	−0.059	0.008	0.50	8.543	0.147	0.011	0.43
Posture and gait disturbances	1.925	0.044	0.046	0.1	7.011	−0.212	0.024	0.23	3.096	0.045	0.023	0.25	2.859	0.027	0.017	0.36	3.463	0.012	0.001	0.85
Kinetic functions	3.89	0.011	0.001	0.84	18.758	−0.893	0.108	0.01	2.151	0.197	0.109	0.01	6.985	−0.101	0.05	0.09	4.024	0.028	0.001	0.82
Speech disorders	0.297	0.006	0.006	0.57	3.963	−0.213	0.175	0.001	0.156	0.033	0.088	0.02	0.838	−0.012	0.020	0.28	0.178	0.035	0.037	0.14
Oculomotor disorders	0.874	0.018	0.015	0.36	2.924	−0.084	0.007	0.53	1.504	0.006	0.001	0.84	1.065	0.019	0.013	0.39	0.81	0.077	0.047	0.1
Neurological soft signs																				
NES Total score	13.154	0.171	0.036	0.15	35.181	−0.964	0.026	0.22	13.787	0.53	0.166	0.001	22.262	−0.101	0.011	0.44	14.649	0.502	0.057	0.07
Sensory integration	2.245	0.008	0.003	0.70	5.996	−0.214	0.046	0.10	1.651	0.081	0.137	0.004	3.534	−0.038	0.051	0.08	1.651	0.09	0.065	0.05
Motor coordination	2.752	0.029	0.012	0.41	8.565	−0.292	0.028	0.20	2.339	0.137	0.128	0.005	5.022	−0.045	0.024	0.24	3.118	0.073	0.014	0.37
Sequencing of complex motor acts	2.076	0.063	0.05	0.09	8.655	−0.26	0.019	0.29	2.852	0.146	0.128	0.005	4.683	−0.009	0.001	0.83	3.035	0.144	0.048	0.09
Psychosocial functioning																				
FAST total score	9.125	0.315	0.063	0.05	40.419	−1.202	0.021	0.27	18.414	0.233	0.017	0.33	14.452	0.245	0.032	0.17	15.585	0.547	0.035	0.15
Autonomy	1.142	0.044	0.033	0.17	5.783	−0.185	0.014	0.38	2.179	0.055	0.025	0.23	2.238	0.021	0.006	0.56	2.325	0.047	0.007	0.53
Occupational functioning	0.999	0.107	0.076	0.03	9.191	−0.259	0.01	0.44	2.905	0.192	0.118	0.007	4.548	0.017	0.002	0.76	2.299	0.276	0.093	0.02
Cognitive functioning	3.39	0.047	0.022	0.26	8.899	−0.23	0.012	0.41	5.243	−0.007	0	0.91	3.545	0.061	0.03	0.19	4.503	0.068	0.008	0.49
Financial issues	1.252	0.015	0.008	0.50	6.327	−0.279	0.062	0.06	1.904	−0.008	0.001	0.80	1.212	0.023	0.015	0.35	1.064	0.077	0.037	0.14
Interpersonal relationships	1.719	0.071	0.036	0.14	5.773	−0.086	0.001	0.79	4.643	−0.024	0.002	0.74	1.746	0.099	0.059	0.06	4.39	−0.001	0	1
Leisure time	0.623	0.031	0.049	0.09	4.446	−0.163	0.031	0.18	1.54	0.024	0.014	0.38	1.163	0.024	0.024	0.24	1.004	0.081	0.06	0.06

BD, bipolar disorder; FAST, Functioning Assessment Short Test; ICARS, International Co-operative Ataxia Rating Scale; NES, Neurological Evaluation Scale; SD, standard deviation.

**Figure 2:**
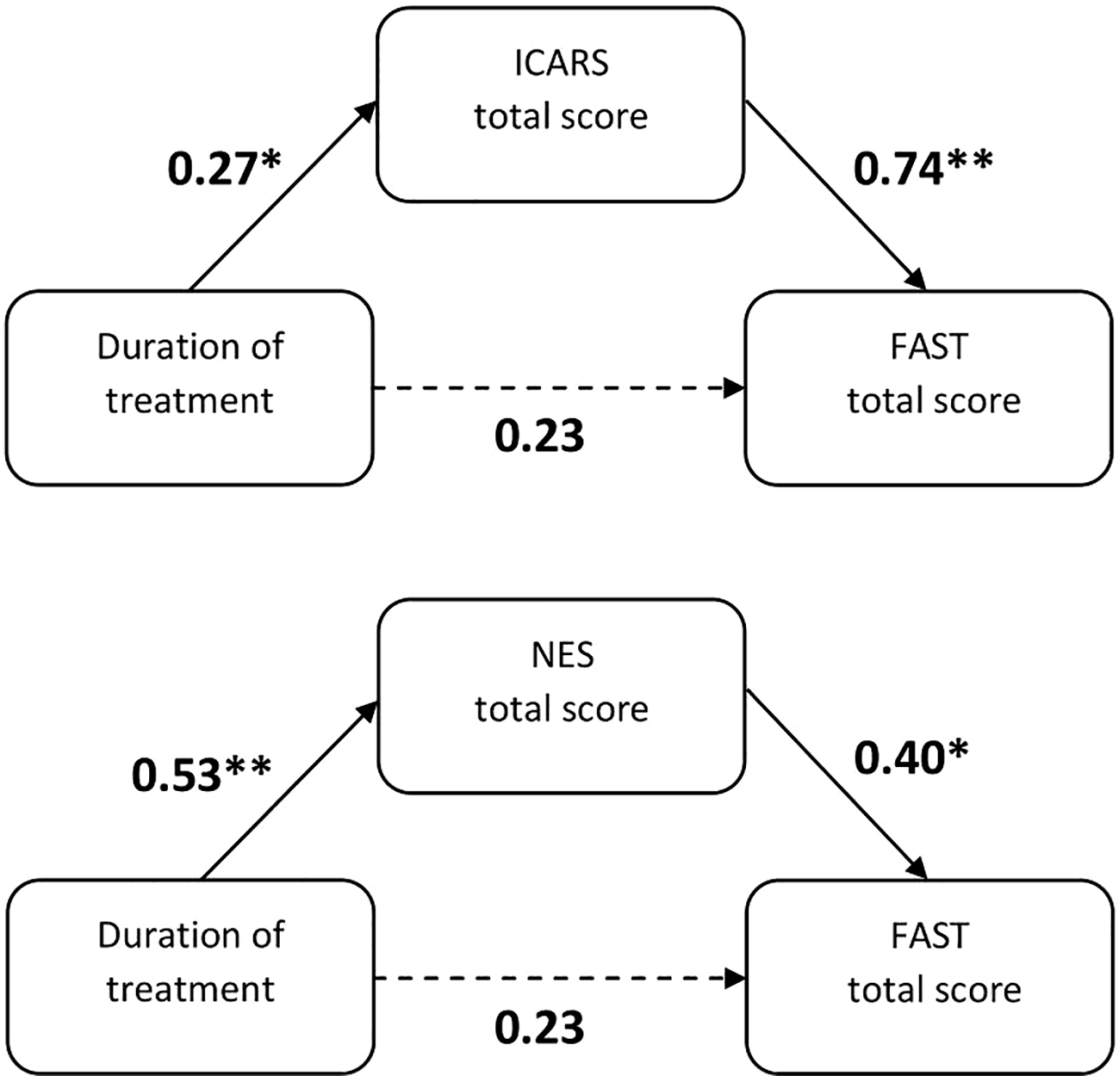
Results of mediation analysis evaluating associations between duration of treatment (independent variable) and FAST total score (dependent variable) through ICARS and NES total scores in the group of patients with BD. The effect of the duration of treatment on FAST total score was fully mediated via the total ICARS and NES scores. Values represent β coefficients of simple linear regression analyses between variables. FAST, Functioning Assessment Short Test; ICARS, International Co-operative Ataxia Rating Scale; NES, Neurological Evaluation Scale. *p ≤ 0.05, **p ≤ 0.01.

We have identified significant associations between the duration of treatment and neurological abnormalities, along with relationships between CSS, NSS, and FAST scores. Consequently, we conducted mediation analysis to assess the associations among these measures. We have shown that rates of NSS and CSS, reflected by total ICARS and NES scores, were full mediators of the relationship between duration of the treatment and psychosocial functioning. Results of the mediation analysis are presented in **Figure 2**; **Table 5**.

**Table 5:** Mediation analysis evaluating associations between duration of treatment (independent variables) and FAST total scores (dependent variable) through ICARS and NES total scores in the group of patients with BD.

Duration of treatment					
Through the total ICARS score					
		Estimate	Lower 95% CI	Upper 95% CI	*p*
FAST total score	ACME	0.2021	0.0142	0.47	0.02
	ADE	0.0304	−0.4046	0.45	0.91
	Total effect	0.2325	−0.1709	0.62	0.25
	ACME/Total effect	0.8692	−5.8609	6.07	0.26
Through the total NES score					
		Estimate	Lower 95% CI	Upper 95% CI	*p*
FAST total score	ACME	0.2122	0.0139	0.42	0.04
	ADE	0.0204	−0.3929	0.57	0.88
	Total effect	0.2325	−0.1554	0.68	0.20
	ACME/Total effect	0.9124	−4.9276	10.06	0.24

FAST, Functioning Assessment Short Test; ICARS, International Co-operative Ataxia Rating Scale; NES, Neurological Evaluation Scale; CI, confidence interval; ACME, average causal mediation effects; ADE, average direct effect.

## 4 Discussion

To the best of our knowledge, this is the first study to evaluate associations between severity of both CSS and NSS and psychosocial functioning in patients with BD. We found small yet significant relationships between these measures. Among neurological deficits, overall severity of CSS was the most significant factor predicting general psychosocial functioning. This measure was also slightly influenced by speech disorders, total rates of NSS, sensory integration deficits, and motor coordination impairments. Our results align with studies demonstrating an association between NSS severity and lower global assessment of functioning and FAST scores in this clinical group (17, 19). In our study, we have analyzed for the first time the impact of neurological abnormalities on specific dimensions of psychosocial functioning. We have found that motor dysfunctions are linked to the patient’s autonomy, decreasing with CSS severity, particularly with kinetic dysfunctions and, to a lesser extent, speech disorders. In contrast, NSS were not associated with the autonomy scores. Occupational functioning subscale has shown the most significant relationship with the severity of both CSS and NSS. Our results corroborate with the observations carried out on the groups of patients with schizophrenia, indicating that NSS are associated with lower occupational status (33) and social class (34). However, several studies have shown no associations between socio-economic status and NSS (35–37). Far fewer studies have assessed these relationships in the group of patients with BD. It has been shown that more severe NSS are associated with poor quality of dwelling and social disability in this clinical group (15, 16).

Previous reports suggested associations between NSS, cognitive impairments (executive functions, verbal, and visual memory), and psychosocial functioning (15, 17). In our study, FAST cognitive functioning subscale showed small, but significant associations with motor coordination deficits measured by NES. Studies performed in the group of patients with schizophrenia indicated that those symptoms were specifically related to impairments in action, attention inhibition, verbal performance, and visual spatial memory (3, 38–40). In our previous study, we have shown that NES motor coordination scores are associated with implicit learning impairments. However, their impact on patients’ psychosocial functioning has not been explored (41).

Financial issues and interpersonal relationships measures were not linked to the neurological abnormalities scores. A single study has shown the opposite, indicating positive association between NES total score and single marital status (42). Further studies are needed to elucidate the role of associations between those measures.

At present, it is difficult to determine the cause of the possible relationships between NSS/CSS and psychosocial functioning. One of the explanations may be the direct influence of the neurological abnormalities on the patients’ daily functioning. The NSS and CSS evaluated in this study comprise a diverse group of symptoms that may influence the patient’s functioning, depending on their daily activities. Joyce et al. (43) have performed a study evaluating the associations between responses to semi-structured interviews capturing changes in the quality of life and daily functioning, as a result of having cerebellar ataxia (43). Patients’ responses on how ataxia symptoms affect their lives were consistent with neurological impairments measured by the four domains of ICARS during clinical examination. For instance, difficulties in speaking at adequate speeds and precision were aligned with the speech disorders score. Ataxia symptoms have also been linked to specific aspects of psychosocial functioning; for example, posture and gait impairments have been shown to correlate with the inability to complete household duties or responsibilities. Although patients with BD do not show neurological abnormalities as severe as those with cerebellar ataxia, the combination of symptoms from different domains (e.g., oculomotor deficits and speech disorders) may impact their psychosocial functioning.

NSS and CSS could also be indirectly related to decreased functionality scores, as they may reflect impairments of the brain structures related to multiple functions. Metanalysis of functional and structural magnetic resonance imaging (MRI) studies indicated that the presence of NSS is most likely a manifestation of cerebello-thalamo-prefrontal brain network impairments (6). The cerebellum has been identified as an important part of this brain circuit. Both structural and functional deficits of those structures are associated with NSS (6, 44, 45). Traditionally, the cerebellum has been viewed as a structure primarily associated with motor functions. However, a growing number of studies changed the view on its role. It has been shown that the cerebellum is exceptionally involved in the control of cognitive functions and affect (46–50). Studies pointed out that this structure may play an important role in pathophysiology of various psychiatric disorders (7). In schizophrenia, it has been hypothesized that disruptions of the cerebellum and its connections with thalamus and prefrontal cortex are associated with “cognitive dysmetria”, consisting in interruption of the fluid execution of mental activities leading to cognitive impairments and clinical symptoms (51). Consistent with this concept, Ho et al. (52) have shown that the presence of the cerebellar signs is associated with poorer cognitive performance, more severe negative symptoms, worse premorbid adjustment, and smaller cerebellar tissue volumes (52). Our findings showing associations between psychosocial functioning measures and CSS align with neuroimaging study results. It has been shown that during BD progression, which is associated with worse inter-episode functioning, there is a significant decrease of cerebellar white matter density and vermal atrophy (53–56). Our previous study indicated that neurological impairment may increase during progression of BD. We have shown that both NSS and CSS are more pronounced in the late stage of the disease in comparison to its early phases. In addition, the severity of most of these motor impairments revealed the association with the duration of treatment (14). In this study, we have shown that both NSS and CSS fully mediate the relationship between treatment duration and patients’ general functioning, as measured by total FAST scores. It is plausible to hypothesize that NSS and CSS severity may reflect structural brain abnormalities associated with BD progression and functional outcome. It can alternatively be hypothesized that the severity of NSS and CSS may indicate neurodevelopmental deficits associated with poorer psychosocial functioning. In this context, measures of motor dysfunctions should correlate with an earlier onset of the disorder. However, our study has demonstrated no significant associations between age of onset and any of the CSS, NSS, and psychosocial functioning measures. Therefore, our findings suggest that the observed correlations between neurological abnormalities, duration of treatment, and FAST scores may be attributed to neuroprogression rather than neurodevelopmental factors. Nevertheless, further research is required to draw more definitive conclusions.

Our studies suggest that in clinical practice, NSS and CSS could serve as accessible biomarkers for BD severity that correspond to patients’ psychosocial functioning independently of the daily dose of antipsychotic medication (14). Previous research has demonstrated the clinical utility of NSS and CSS assessments primarily in patients with schizophrenia (3). The evaluation of NSS has been proposed to help identify patients with more pronounced neuropathology, potentially predisposing them to a more chronic and severe course of illness (3). Future research on the clinical utility of neurological abnormalities assessment in BD can draw inspiration from these earlier studies. The use of CSS and NSS examinations in clinical practice necessitates support from longitudinal studies that assess the relationships between their severity and the psychosocial functioning, clinical outcomes, and structural and functional brain changes in patients with BD (14).

Although there is a considerable body of literature on neuroimaging in BD, research linking structural brain disturbances to patient functioning measures remains limited. Previous studies have not assessed the relationships between these disturbances and the structural and functional impairments of the cerebellum and its neuronal network. In the area of schizophrenia research, Wassink et al. (57) have shown that smaller cerebellar volume is associated with more severe psychosocial deficits (57). Further neuroimaging studies are required to evaluate relevance of our findings.

Our study is not free of various limitations. We have not used scales evaluating movement disorders induced by antipsychotics. In addition, we have excluded patients with substance abuse; however, we did not assess the frequency of alcohol consumption in the studied groups. We are aware that the relationship between measures of neurological abnormalities and psychosocial functioning is relatively small, albeit statistically significant. The measures of the daily functioning of patients with BD will be influenced by many factors with a great number of unexplainable variations. The small effect size of those relationships corresponds with the results of Baş et al. (2015) (17). Authors showed inverse correlations between NSS severity and functioning scores and cognitive impairments. However, regression analyses showed that total NES scores do not increase the model that predicted total FAST score, which was mostly dependent on verbal memory deficits and subsyndromal symptoms. Further studies are required to evaluate the relevance of the associations between CSS, NSS, and patients’ psychosocial functioning. The generalizability of our results may be limited due to the relatively homogeneous sample of patients with BD. Future studies should consider including more diverse BD populations, such as patients on lithium or other mood stabilizers. The strength of our study is the fact that the rater assessing neurological abnormalities was blind to patient’s FAST scores, BD type, and history of psychotic symptoms.

## 5 Conclusions

Our results point out that both NSS and CSS severity shows small but significant associations with the general psychosocial impairments of patients with BD. Aspects of the daily functioning related to these neurological abnormalities are occupational status and autonomy measures. Moreover, our results suggest that NSS and CSS may mediate associations between BD progression and psychosocial deficits. We have shown that NES and ICARS total scores were a full mediator of the relationship between duration of the treatment and total FAST scores. Our results suggest that even “soft” neurological abnormalities may have an impact on the psychosocial functioning of patients with BD.

## Data Availability

The raw data supporting the conclusions of this article will be made available by the authors, without undue reservation.
